# Bridging the Gap: Translated Medical Education to Support Cystic Fibrosis Centers From Non‐English Speaking Countries

**DOI:** 10.1002/ppul.71764

**Published:** 2026-07-30

**Authors:** Chris Smith, Helen K. Chadwick, Daniel G. Peckham, Kate Hill, Oana Voivod, Marko Krasnyk, Yasemin Gökdemir, Ilknur Gorgun, Fulya Özdemircioğlu, Halyna Makukh, Hilde De Keyser, Claire Francis, Katarina Štěpánková, Csilla‐Eniko Szabo, Pavel Drevinek

**Affiliations:** ^1^ Royal Alexandra Children's Hospital Brighton UK; ^2^ Brighton and Sussex Medical School Brighton and Hove UK; ^3^ Leeds Teaching Hospitals NHS Trust Leeds UK; ^4^ Leeds Institute of Medical Research University of Leeds Leeds UK; ^5^ NI Clinical Research Facility, WWIEM Queen's University Belfast UK; ^6^ ECFS CTN Karup Denmark; ^7^ Together for Patrick Association Cluj‐Napoca Romania; ^8^ ECFS Patient Registry Service Desk Switzerland; ^9^ Division of Pediatric Pulmonology School of Medicine Marmara University Istanbul Türkiye; ^10^ KIFDER Istanbul Türkiye; ^11^ Ivan Franko National University of Lviv Lviv Ukraine; ^12^ Cystic Fibrosis Europe Brussels Belgium; ^13^ Slovenská Asociácia Cystickej Fibrózy Košice Slovakia; ^14^ Pediatrics I Discipline, Mother and Child Department “Iuliu Hațieganu” University of Medicine and Pharmacy Cluj‐Napoca Romania; ^15^ Pediatrics 1 Clinic Clinical Emergency Hospital for Children Cluj‐Napoca Romania; ^16^ Department of Medical Microbiology, Second Faculty of Medicine Motol and Homolka University Hospital, Charles University Prague Czech Republic

**Keywords:** artificial intelligence, ECFS, education, translation

## Abstract

**Background:**

The European Cystic Fibrosis Society (ECFS) develops education resources to support members; however, these are almost exclusively in English. Many barriers to translation exist, including cost and time. Artificial intelligence (AI) provides an opportunity to support translation and address such barriers. This study aimed to pilot the use of AI‐generated translation of ECFS e‐learning modules and evaluate the quality.

**Methods:**

An AI translation program was used to create subtitles of ECFS peer‐reviewed education modules. Two independent native language speakers with extensive cystic fibrosis (CF) healthcare experience were identified and tasked with reviewing, editing, and validating. This was followed by the development and circulation of an online evaluation survey assessing users' views on quality.

**Results:**

Education packages, each consisting of six subtitled modules, were created in three languages: Ukrainian, Romanian, and Turkish. For each language, corrections to the AI‐generated translation by the independent native speakers were essential. Evaluation was conducted in two countries. Eighteen completed surveys were received. Results indicated high levels of accuracy for the final modules, and feedback was very positive regarding the utility and range of topics.

**Conclusion:**

The use of novel AI‐generated translation shows promise and proved quick and affordable. However, quality of translation was variable, highlighting the critical role of collaborating with native‐speaking CF experts to ensure linguistic accuracy. This project highlights the importance of interdisciplinary collaborative efforts between ECFS Education, the Twinning Project, CF Europe, and patient organizations. Further, it demonstrates both the feasibility and practicality of generating effective multilingual educational modules using AI.

## 1 Introduction

### 1.1 The Society

The European Cystic Fibrosis Society (ECFS) is an international organization of researchers and clinical professionals. It is committed to improving survival and quality of life for people with cystic fibrosis (CF) through the promotion of high‐quality research, education, and care.

To support the Society's mission, the ECFS Education Committee (ECFS‐EC) was established in 2016 with responsibility for developing and delivering education. The Society provides a wide range of educational opportunities for its members in multiple formats, including international conferences, regular webinars, and structured syllabuses [1]. One specific initiative is the e‐Learning Project, which involves the development of a collection of concise, peer‐reviewed, video modules delivered by international experts covering a range of aspects of CF care. While this—and all education delivered by ECFS—are highly valuable, they are provided almost entirely in English, which limits accessibility and utility for some members of the CF community.

### 1.2 The Twinning Project

The ECFS/CF Europe “Twinning Project” is an initiative designed to foster strong partnerships and lasting relationships between CF communities across different countries [2]. Briefly, the project pairs an expert CF center with a long‐standing record of excellence in CF care (the “mentor site”) with a “mentee site”, a center aiming to improve clinical outcomes through guidance, collaboration, and shared expertise. In addition, it pairs well‐resourced patient organizations with advanced advocacy, digital expertise, and fundraising capabilities to patient organizations needing more support or training. This project addresses recognized service gaps [3] and responds to a recent international survey indicating insufficient training opportunities for healthcare professionals in Eastern Europe [4]. One original objective of the project was to support mentee sites by providing open access to the ECFS Education Platform, which houses the Society's high‐quality education content. However, providing access alone does not ensure comprehension.

### 1.3 Artificial Intelligence (AI)

AI use has expanded rapidly across many domains of healthcare and has been widely evaluated [5]. One such promising area is medical education [6, 7] and, more specifically, in the translation of resources [8]. Historically, translation of any medical education has been limited by time, cost, and resource constraints‐barriers that AI has the potential to overcome.

These three areas—ECFS educational content, the identified needs of under‐resourced countries, and the emerging potential of AI‐generated translation—intersect to create a unique and timely opportunity to enhance education accessibility and strengthen CF care globally.

The educational aims of this initiative within the Twinning project were twofold. First, to pilot the use of AI‐generated translation of ECFS e‐learning modules into three languages spoken in mentee countries (Ukraine, Romania, and Türkiye). Second, to evaluate the quality and usability of translated modules in two of these languages (Ukrainian and Romanian).

## 2 Methods

Scoping was undertaken to understand the specific considerations for translation of medical educational content. A commissioned 2016 report from the European Centre for Disease Prevention and Control, which describes a five‐step, stakeholder‐based approach to adapting health communication materials [9], was identified and used as a framework (Table 1).

**Table 1:** 5‐Step cultural adaptation of health communication materials [9].

1	Careful selection of materials and process coordinators
2	Early review by content and linguistic experts
3	Translation and quality check
4	Comprehension testing
5	Proofreading, design, networking, and evaluation

An AI‐based translation service (www.Sonix.ai) was identified. Each module from the e‐Learning Project comprised of an approximate 20 min peer reviewed PowerPoint lecture with audio discussion, clear learning objectives, take‐home messages, and references. Six modules—Epidemiology; Diagnosis; Overview of clinical symptoms; Principles of respiratory care; Nutrition, liver, and diabetes; and Psychosocial—were uploaded to the service. Sonix was used to transcribe each video module to produce an initial transcription and subtitles in English. The subtitles were then reviewed to ensure accuracy and correct timing by two English authors. After selecting the target language, Sonix generated an AI‐based translation of the English subtitles. Each module with the translated subtitles were made accessible via the web‐based interface to a native‐speaker reviewer for checking and editing. Reviewers watched the videos while listening to the English audio and corrected any errors in the translated subtitles. Corrections such as grammatical errors could be made within the subtitles directly and adjustments to timings made as the videos played. Following completion by the first reviewer, access was provided to a second reviewer for cross‐checking and confirmation of the accuracy of all aspects. Once approved by both native language reviewers, the ECFS‐EC embedded the subtitles into the modules and compiled them into a six‐module “Education Package” (EP). This process was then repeated for each additional language, following the same steps and using the reviewers identified for that language.

The Twinning Project Steering Team identified and provided two reviewers for each language. For each country, a minimum of one current CF physician was required, with a second reviewer potentially drawn from a national CF patient organization with current lived experience of CF. Online meetings were arranged with each reviewer to discuss and clarify project background, technology use, and task scope. Reviewers were tasked with correcting medical and grammatical errors and ensuring cultural appropriateness. The project used subtitles and is therefore classified as audiovisual translation, which converts spoken dialogue into written text, unlike interpretation, which occurs in real time.

Two international events attended by Romanian and Ukrainian CF health professionals (physicians, multidisciplinary team members, and medical students) (Southeastern 2024 CF Conference in Romania and the V4‐CF 2024 conference in Poland), coincided with the project. This opportunity was used to pilot distribution and evaluation of the EPs. USBs were preloaded with the EPs and disseminated to participants in person at the events by the ECFS‐EC. Contact information was collected to allow for follow‐up evaluation. An online evaluation survey (SurveyMonkey.com) was developed by ECFS‐EC using a 7‐point Likert scale and translated into the relevant language by the reviewer team. The survey was distributed to the collected email addresses. Each email contained a shareable link to access and complete the survey. The initial invitation was sent to 28 healthcare professionals in Ukraine and 11 in Romania. Recipients were asked to forward the survey link to any healthcare professionals who had viewed the e‐learning modules, rather than restricting participation to the original recipients. The survey questions were designed to elicit information on the following:

i. The usefulness and quality of translated subtitles in helping learners understand the module;
ii. Whether the subtitles were synchronized with the slide pace;
iii. Whether the module improved understanding and expertise;
iv. Whether participants would find additional translated educational materials helpful.

Ethical approval was not required for this project. Study details were entered into the UK Health Research Authority online decision tool, which determined that the work did not constitute research and therefore did not require formal ethics review. As a service evaluation, with formal ethical approval confirmed as not required, participants were provided with information, and consent was implied by completion of the questionnaire. In addition, data were anonymized; therefore, individual consent would not be required. This was compliant with the SQUIRE checklist.

## 3 Results

Three language‐specific EPs, each consisting of six subtitled modules, were produced for Ukraine, Türkiye, and Romania. The quality of initial translation by Sonix varied between countries. For each language, all AI‐generated modules required corrections by the independent native speakers. Corrections were required in all domains: spelling, grammar, semantics, cultural appropriateness, and consistency.

**Figure 1:**
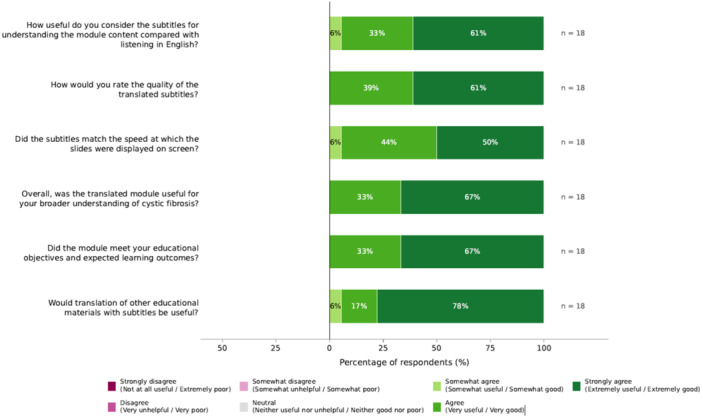
Participant evaluation ratings of education packages presented as a diverging stacked bar chart of Likert‐scale responses. [Color figure can be viewed at wileyonlinelibrary.com]

Responses from 18 participants were received (6 from Romania and 12 from Ukraine). As the total number of eligible individuals who received the survey invitation was unknown, a total response rate could not be calculated. On the 7‐point Likert scale (1 = *strongly agree*; 7 = *strongly disagree*), 94% of respondents considered the subtitles as either extremely useful or very useful (scores 1 or 2) for understanding the module content compared to listening to it in English. One hundred percent of responders rated the quality of the subtitle translations either extremely good (61%) or very good (39%) (scores 1 or 2). Ninety‐four percent either totally agreed or agreed (scores 1 or 2) that the subtitles matched the speed of the slides. One hundred percent of respondents either totally agreed (67%) or agreed (33%) (scores 1 or 2) that the translated module was very useful for improving understanding and expertise in CF. One hundred percent either totally agreed (67%) or agreed (33%) (scores 1 or 2) that the module met the educational objectives and the expected learning outcomes. Ninety‐five percent totally agreed (78%) or agreed (17%) (scores 1 or 2) that it would be useful to translate other educational materials with subtitles. Results are illustrated in Figure 1. Results suggested high levels of accuracy of the final EPs, and feedback was very positive regarding the utility and range of topics.

## 4 Discussion

Health inequality gaps in CF continue to be reported [10, 11]. Specifically, a broad body of work has described the significant inequalities in provision of continued medical education/continued professional development (CPD) in low middle‐income countries (LMIC) [12] that may contribute to this. The ECFS has a responsibility to support all members in countries of all income levels by disseminating knowledge across languages and resource settings, to optimize patient care and support CPD [13]. This is not just a broad or abstract responsibility; a 2023 international survey of the CF health provider community demonstrated a clear demand for educational materials [14]. Furthermore, translated education material emerged as the highest priority in non‐English‐speaking countries.

To address this challenge, we present a structured, AI‐assisted approach to translating educational materials, highlighting the collaborative efforts and experiences involved.

## 5 Considerations

### 5.1 Accuracy

While translation of educational material into local languages offers many benefits, it also introduces challenges and risks, leading to important practical and conceptual lessons. There is an emerging focus on both the positive potential and limitations of AI in education [15, 16]. A recent review identified and listed many available AI translation tools, including the one used in this initiative [17]. A consistent concern regarding the use of AI‐based translation is the variability in accuracy, which introduces the risk of critical and misleading translation errors [17]. Such errors may acutely affect care and, through misinformation, cause long‐term harm to clinical decision‐making and professional teaching. A recent systematic review focused on the accuracy, acceptability, and usability of language translation performed by AI in the clinical setting. Even with high reported accuracy (e.g., Google Translate at 92.2%) [18], minor precision losses may negatively affect clinical decision‐making, underscoring the need for strict oversight. The integration of human oversight—a term now coined as “human‐in‐the‐loop” is becoming more established and supports this approach to facilitate reliable, effective, and equitable use of AI translation in clinical practice [19].

In addition to basic spelling or grammatical accuracy errors, broader errors resulting from more subtle cultural aspects are also recognized. Translation is not limited to language conversion alone; the concept of cross‐cultural contextualization and adaptation must consider both language translation and cultural relevance if the original intent and educational value of the content are to remain. At present, AI's ability to do this is limited, and this essential part of ensuring clinical accuracy and contextual appropriateness requires resource‐heavy human expertise and nuance. In our experience, all reviewers had to make multiple changes and edits to the original translated subtitles. In some cases, this could take considerable time. In addition to this, an unexpected challenge was that subtitle timing could be difficult, as the number of words or letters often differed between languages, making it challenging to ensure that subtitles remained synchronized with the visuals on the PowerPoint slides.

### 5.2 Cost

Cost has long been a significant barrier to translation efforts. Although AI shows promise in translation and may potentially alleviate some costs [17], human oversight remains essential and, therefore, cost considerations (although less) persist. This issue has been widely examined, especially regarding responsibility and, most controversially, who should bear the financial burden. Options proposed include providers, insurance, patients, volunteers, and governments [20].

Multilingual physicians may be considered essential to lead such initiatives, as they have the specific expertise and depth of knowledge required. Many attend international conferences, so levels of English are likely to be high through exposure to research and professional meetings. However, clinicians in resource‐limited settings often carry large caseloads and may lack the support available in better‐resourced centers [4].

Patient participation could also be considered mandatory. Potential benefits include faster implementation and earlier impact compared with waiting for infrastructure funding. There is also the advantage that the work may benefit not just one patient, but future patients, clinics, or entire communities. This approach can be empowering, as patients who invest in their care are often more engaged and may strengthen their relationship with clinicians. However, potential drawbacks include conflicts of interest, insufficient knowledge of medical terminology, ethical concerns, and blurred professional boundaries.

Finally, patient organizations are another potential contributor. Their strengths include strong advocacy, enthusiasm for practical improvements, and the ability to mobilize funding and volunteers rapidly, particularly for urgent and highly relevant education initiatives.

However, some of these groups may lack medical expertise, which can lead to oversimplification if not appropriately guided.

For this project, ECFS/CF Europe Twinning project funding covered the highest cost, the Sonix license. However, this may not be sustainable in the long term. A collaborative model to address responsibility and cost is preferable. Such an approach would not only help achieve the desired outcomes but also promote collaboration, ensure shared responsibility, and foster a shared sense of achievement.

### 5.3 Lack of Agreed International Methodology

Medical education translation lacks a consistent international standard, which is a major limitation. However, guidance and best‐practice recommendations do exist that can provide a framework. European recommendations have been proposed that formed the basis of our work [9], but other notable elements that have been described were also applied. For example, Garcia‐Castillo and Fetters described four elements: (1) involving experts with a high level of fluency in the two languages involved; (2) utilizing content experts in the material that is being translated; (3) steadfast attention to cultural equivalence in the formulation of the final translation; and (4) recording an accessible documentation trail [20].

### 5.4 Evaluation Sample Size

A limitation of the evaluation was the relatively small sample size of responders. However, the survey targeted a highly specific group within a rare condition, in countries where resources and specialist clinicians are limited, underscoring the purpose of the whole project. In addition, the CF European Registry highlights that patient numbers in Romania and Ukraine (416 and 508, respectively) are small compared with larger Western centers [21]. Although the overall response rate was relatively low, it likely represents a meaningful proportion given the small, targeted pool of experts working in this area. A further limitation is the absence of data from Türkiye, where no evaluation data were collected. Although this limited the comprehensiveness of the evaluation, future studies should ensure that feedback is obtained from every country in which the process is implemented. We intend to collect evaluation data from Türkiye in future work, now that the educational partnership has been established. The available data remain valuable for early‐stage evaluation, as this study was exploratory and pilot in nature, with findings intended to inform the future development of the initiative.

### 5.5 Strengths

Despite the highlighted considerations (errors, costs, and lack of an international methodology for medical education translation), it is clear that translating medical education provides substantial benefits. These include equitable access to training, improved comprehension, enhanced clinical practice, reduced disparities in care delivery, and improved patient safety. Importantly, translated resources support CPD which is recognized as a critical component of CF care [22].

A key strength was the use of existing high‐quality, peer‐reviewed educational modules developed under ECFS oversight, maximizing available resources and extending international reach. This ensured high‐quality education and reduced the risk of clinicians relying on unregulated AI tools for translation. A major strength was the involvement of a “human‐in‐the‐loop” and that these were experts with deep, current knowledge of CF, ensuring condition‐specific accuracy. In addition, the native speaking experts were engaged from a combination of mentee sites and patient organizations, drawing on the respective advantages of each, as described above, and exemplifying collaborative working towards a common goal.

Similar work has illustrated the critical role of culturally competent researchers in reviewing transcripts to ensure accuracy and clarity [20, 23]. This extends beyond just the modules themselves to the translation of the evaluation surveys, which was essential to ensure a clear understanding and to generate reliable, high‐quality feedback.

A recent systematic review was favorable to offline digital education [24] and specifically USB's have been described as a method to support and address inequalities in CPD in LMIC [25]. Using USB drives to distribute the translated EPs not only removed the language barrier but also addressed several other practical barriers. USBs eliminate the need for passwords or logins. They work without reliable internet, avoid website access issues (such as permissions on work computers), provide instant access without long downloads of large files, and can be easily shared between internal departments. In addition, the EPs are highly flexible, allowing learners to tailor them to their individual needs and to review, repeat, or resume the material as required. However, while these resources enhance accessibility, this comes with the limitation that their reach and impact cannot be fully evaluated, as it is not possible to determine how often they are accessed, by whom, or the extent to which they are shared.

Finally, this initiative is in strong alignment with the WHO's Global Strategy on Digital Health framework, which promotes the use of digital tools in healthcare, considering cost‐effectiveness, evidence‐based, and patient engagement [26]. More specifically, it addresses training and education—identified as key building blocks for improving global CF care [27]. The collaboration also advances the goal of partnering those healthcare providers in LMIC settings with providers who have educational expertise [28].

## 6 Conclusion/the Future

This project illustrates how emerging technologies can help overcome educational barriers as well as the complexity and steps needed to successfully deliver accurate multilingual modules. Evaluations of the resulting quality of educational material were positive and support the further expansion of the translation of other modules and educational resources. It promotes partnerships and highlights collaborative working between the ECFS, the Twinning project, CF Europe, and local patient organizations and communities. Further work is already underway, applying the principles and lessons learned in this initiative in Albania, Armenia, Latvia, and Georgia. Future planning will involve offering a more bespoke approach to the selection of key topics. Although these resources were developed primarily for healthcare professionals, they were made openly accessible without restricting the intended audience, recognizing that patients, families, and the wider community may also benefit. The development of resources specifically informed by patient and family perspectives represents a potential future direction for this project. Such work would require a clearer understanding of the needs and preferences of these groups and may need to be tailored to different healthcare settings.

When done well, this process will improve accessibility to learning material, improve clinical practice, address inequalities, and foster collaboration. Addressing linguistic barriers is essential to improve the accessibility and implementation of educational resources across Europe, where more than 20 official languages are spoken. Although this work was undertaken within a European context, the challenges it addresses are not unique to Europe. Gaps in access to high‐quality medical education persist globally, particularly in LMICs, suggesting that the principles demonstrated in this project may be transferable to other healthcare settings. AI‐generated translation shows promise as a quick and affordable option; however, our experience underscores that collaboration with local CF experts and rigorous oversight remain essential.

## Author Contributions

**Chris Smith:** conceptualization, formal analysis, methodology, supervision, project administration, writing – review and editing, writing – original draft, visualization. **Helen K. Chadwick:** conceptualization, formal analysis, methodology, project administration, visualization, supervision, writing – review and editing. **Daniel G. Peckham:** conceptualization, supervision, writing – review and editing. **Kate Hill:** formal analysis, methodology, writing – review and editing. **Oana Voivod:** methodology, writing – review and editing. **Marko Krasnyk:** methodology, writing – review and editing. **Yasemin Gökdemir:** methodology, writing – review and editing. **Ilknur Gorgun:** methodology, writing – review and editing. **Fulya Özdemircioğlu:** methodology, writing – review and editing. **Halyna Makukh:** methodology, writing – review and editing. **Hilde De Keyser:** methodology, writing – review and editing. **Claire Francis:** methodology, writing – review and editing. **Katarina Štěpánková:** methodology, writing – review and editing. **Csilla‐Eniko Szabo:** methodology, writing – review and editing. **Pavel Drevinek:** conceptualization, visualization, supervision, writing – review and editing.

## Conflicts of Interest

Chris Smith: Has received advisory fees from Vertex Pharmaceuticals and Nordic Pharma and speaker fees from Vertex Pharmaceuticals. Daniel G. Peckham: Has received speaker/board honoraria from Vertex Pharmaceuticals. Pavel Drevinek: Has received speaker fees from Vertex Pharmaceuticals. The other authors declare no conflicts of interest.

## Data Availability

The data that support the findings of this study are available from the corresponding author upon reasonable request.

## References

[1] C. Smith , H. K. Chadwick , N. Shaw , et al., “Development of a Multidisciplinary Syllabus to Support the Education and Training of Roles in Cystic Fibrosis Care: An ECFS Education Initiative,” Journal of Cystic Fibrosis 25, no. 2 (2026): 336–339. 41309412 10.1016/j.jcf.2025.11.012

[2] P. Drevinek , H. K. Chadwick , K. Stepankova , et al., “Partnership Between Cystic Fibrosis Centres in Europe: The Twinning Project,” Journal of Cystic Fibrosis (2026), 10.1016/j.jcf.2026.06.009. 42362443

[3] K. Walicka‐Serzysko , M. Peckova , J. J. Noordhoek , D. Sands , and P. Drevinek , “Insights into the Cystic Fibrosis Care in Eastern Europe: Results of Survey,” Journal of Cystic Fibrosis 17, no. 4 (2018): 475–477. 29681443 10.1016/j.jcf.2018.04.003

[4] A. F. Montiel , A. Á. Fernández , M. C. Amigo , et al., “Standards of Care and Educational Gaps in Adult Cystic Fibrosis Units: A European Respiratory Society Survey,” ERJ Open Research 10, no. 3 (2024): 00065–02024. 38746857 10.1183/23120541.00065-2024 PMC11089383

[5] N. R. Sahni and B. Carrus , “Artificial Intelligence in US Health Care Delivery,” New England Journal of Medicine 389, no. 4 (2023): 348–358. 37494486 10.1056/NEJMra2204673

[6] B. G. Verghese , C. Iyer , T. Borse , S. Cooper , J. White , and R. Sheehy , “Modern Artificial Intelligence and Large Language Models in Graduate Medical Education: A Scoping Review of Attitudes, Applications & Practice,” BMC Medical Education 25, no. 1 (2025): 730. 40394586 10.1186/s12909-025-07321-5 PMC12093616

[7] Z. Ahsan , “Integrating Artificial Intelligence into Medical Education: A Narrative Systematic Review of Current Applications, Challenges, and Future Directions,” BMC Medical Education 25, no. 1 (2025): 1187. 40849650 10.1186/s12909-025-07744-0 PMC12374307

[8] A. Genovese , S. Borna , C. A. Gomez‐Cabello , et al., “Artificial Intelligence in Clinical Settings: A Systematic Review of Its Role in Language Translation and Interpretation,” Annals of Translational Medicine 12, no. 6 (2024): 117. 39817236 10.21037/atm-24-162 PMC11729812

[9] “Translation Is not Enough: Cultural Adaptation of Health Communication Materials,” European Centre for Disease Prevention and Control, 2016. https://www.ecdc.europa.eu/sites/default/files/media/en/publications/Publications/translation-is-not-enough.pdf.

[10] I. Sermet‐Gaudelus , A. Orenti , E. Hatziagorou , et al., “Health Inequity in People With Cystic Fibrosis: Can We Close the Gap?,” Annals of the American Thoracic Society 23, no. 2 (February 2026): 228–240. 40929684 10.1513/AnnalsATS.202501-052OC

[11] J. Guo , I. King , and A. Hill , “International Disparities in Diagnosis and Treatment Access for Cystic Fibrosis,” Pediatric Pulmonology 59, no. 6 (2024): 1622–1630. 38558542 10.1002/ppul.26954

[12] E. Hill , D. Gurbutt , T. Makuloluwa , et al., “Collaborative Healthcare Education Programmes for Continuing Professional Education in Low and Middle‐Income Countries: A Best Evidence Medical Education (BEME) Systematic Review,” Medical Teacher 43, no. 11 (2021): 1228–1241. 34499841 10.1080/0142159X.2021.1962832

[13] J. C. Davies , E. Bakkeheim , A. Chansard , et al., “Perspective of the European Cystic Fibrosis Society on Improving Global Cystic Fibrosis Care,” Pediatric Pulmonology 61, no. 7 (2026): e71720. 42400501 10.1002/ppul.71720 PMC13332699

[14] C. Smith , H. K. Chadwick , K. Hill , and D. G. Peckham , “E‐Learning Within the European Cystic Fibrosis Society: A Multidisciplinary Cross‐Sectional Survey,” Journal of Cystic Fibrosis 23, no. 5 (2024): 1020–1023. 38997825 10.1016/j.jcf.2024.07.003

[15] C. J. Webber , P. Whitworth , and S. P. Stenner , “The Future of Artificial Intelligence in Medical Education and Continuing Medical Education,” Primary Care: Clinics in Office Practice 52, no. 4 (2025): 781–797. 41110916 10.1016/j.pop.2025.07.010

[16] L. Turner , C. Zhou , and J. Burk‐Rafel , “It Takes More Than Enthusiasm: The Missing Infrastructure to Unlock AI's Potential in Medical Education,” supplement, Academic Medicine 100, no. 9S S1 (September 2025): S34–S38. 40456123 10.1097/ACM.0000000000006104

[17] I. S. Karakus , I. Strechen , A. Gupta , et al., “Bridging Language Gaps in Healthcare: A Systematic Review of the Practical Implementation of Neural Machine Translation Technologies in Clinical Settings,” Journal of the American Medical Informatics Association 32, no. 11 (2025): 1756–1766. 40966445 10.1093/jamia/ocaf150 PMC12626213

[18] J. Birkenbeuel , H. Joyce , R. Sahyouni , et al., “Google Translate in Healthcare: Preliminary Evaluation of Transcription, Translation and Speech Synthesis Accuracy,” BMJ Innovations 7, no. 2 (2021): 422–429.

[19] R. C. Brewster , G. Tse , A. L. Fan , et al., “Evaluating Human‐in‐the‐Loop Strategies for Artificial Intelligence‐Enabled Translation of Patient Discharge Instructions: A Multidisciplinary Analysis,” NPJ Digital Medicine 8, no. 1 (2025): 629. 41136708 10.1038/s41746-025-02055-6 PMC12552660

[20] D. Garcia‐Castillo and M. D. Fetters , “Quality in Medical Translations: A Review,” Journal of Health Care for the Poor and Underserved 18, no. 1 (2007): 74–84. 17337799 10.1353/hpu.2007.0009

[21] A. A. A. Zolin and E. Bakkeheim , “ECFSPR Annual Report 2023,” 2025. https://www.ecfs.eu/sites/default/files/Annual%20Report_2023_vs1.2_ECFSPR_20250721.pdf.

[22] S. Conway , I. M. Balfour‐Lynn , K. De Rijcke , et al., “European Cystic Fibrosis Society Standards of Care: Framework for the Cystic Fibrosis Centre,” Journal of Cystic Fibrosis 13 (2014): S3–S22. 24856776 10.1016/j.jcf.2014.03.009 PMC7105239

[23] S. Akramul Kabir , F. Ali , and R. Sulaiman‐Hill , “A Comparative Assessment of AI and Manual Transcription Quality in Health Data: Insights From Field Observations,” New Zealand Medical Journal 138, no. 1625 (2025): 35–43. 10.26635/6965.7024 41197094

[24] B. M. Kyaw , P. Posadzki , G. Dunleavy , et al., “Offline Digital Education for Medical Students: Systematic Review and Meta‐Analysis by the Digital Health Education Collaboration,” Journal of Medical Internet Research 21, no. 3 (2019): e13165. 30907731 10.2196/13165 PMC6452290

[25] J. Pelletier , Y. Li , E. Cloessner , et al., “Bridging Gaps: A Quality Improvement Project for the Continuing Medical Education on Stick (CMES) Program,” Cureus 16, no. 6 (2024): e62657. 39036234 10.7759/cureus.62657 PMC11258952

[26] “Global Strategy on Digital Health 2020‐2027,” World Health Organization, Geneva, 2025. https://iris.who.int/server/api/core/bitstreams/a64d584f-015b-471f-9d86-9ea263cfb516/content.

[27] S. Z. Nasr , S. Fleifil , T. B. Kamel , et al., “Roadmap to Global Cystic Fibrosis Care,” Pediatric Pulmonology 60, no. 11 (2025): e71390. 41267340 10.1002/ppul.71390

[28] S. C. Bell , M. A. Mall , H. Gutierrez , et al., “The Future of Cystic Fibrosis Care: A Global Perspective,” Lancet Respiratory Medicine 8, no. 1 (2020): 65–124. 31570318 10.1016/S2213-2600(19)30337-6 PMC8862661

